# Recent Advances in Nutrition and Diabetes

**DOI:** 10.3390/nu13051573

**Published:** 2021-05-08

**Authors:** Omorogieva Ojo

**Affiliations:** School of Health Sciences, Faculty of Education, Health and Human Sciences, University of Greenwich, Avery Hill Campus, Avery Hill Road, London SE9 2UG, UK; o.ojo@greenwich.ac.uk; Tel.: +44-20-8331-8626; Fax: +44-20-8331-8060

The prevalence of diabetes is on the increase worldwide, being one of the fastest growing international health emergencies in the 21st century. Diabetes that is not well managed can have significant impact on morbidity and mortality, including being a risk factor for stroke, renal dysfunction, leg amputation, cardiovascular diseases, loss of vision, and neuropathy. The economic costs of diabetes can also be quite profound through direct medical costs to individuals, families and healthcare systems, and loss of work and wages. Strategies for managing diabetes often include regular physical activity, smoking cessation, maintaining health body weight, and healthy diet. In addition, nutritional interventions in the management of diabetes may involve reduction of calories in diets, the use of diets with low glycaemic index, and increasing the fibre content of diets.

The role of dietary fibre in health and diseases such as diabetes, cardiovascular disease, colon cancer, and obesity has been the subject of significant interest. While it has been revealed that dietary fibre plays a role in the pathogenesis of chronic diseases, an evolving area of research and attention is its effect on gut microbiota and type 2 diabetes. There is evidence that diets that are high in fat and low in fibre may promote gut microbiota dysbiosis as a result of the loss of beneficial and protective microbes, while a high-fibre diet could inhibit protein fermentation and promote gut microbial eubiosis. Dietary fibre has been reported to be a major energy source for intestinal bacteria and thus can significantly affect the diversity of gut microflora.

Therefore, the current editorial provides an overview of the articles published in the Special Issue on recent advances in nutrition and diabetes. It focuses on the role of diet in the pathophysiology and management of diabetes including its effect on gut microbiota. In this regard, Ojo et al. [1] conducted a systematic review and meta-analysis of randomised controlled trials and sought to evaluate the role of dietary fibre in modulating gut microbiota dysbiosis in patients with type 2 diabetes. The authors relied on the Preferred Reporting Items for Systematic Reviews and Meta-Analyses. Electronic databases including EBSCOHost with links to Health Sciences Research Databases, EMBASE, Google Scholar, and reference lists of articles were searched for relevant studies.

Searches were conducted from date of commencement of the database to 5 August 2020, and these were based on the Population, Intervention, Comparator, Outcomes, Studies (PICOS) framework [1].

There were nine studies which met the inclusion criteria and were selected for the systematic review and meta-analysis. Four distinct areas were identified, namely, the effect of dietary fibre on gut microbiota, the role of dietary fibre on short-chain fatty acids (SCFAs), glycaemic control, and adverse events [1]. The authors found significant difference (*p* < 0.01) in the relative abundance of Bifidobacterium with a mean difference of 0.72 (95% CI, 0.56, 0.89) total SCFAs (*p* = 0.02), with a standardised mean difference of 0.5 (95% CI, 0.08, 0.91) between the dietary fibre group compared with placebo. However, there were no significant differences (*p* > 0.05) between the groups with respect to acetic acid, propionic acid, and butyric acid [1].

Furthermore, there was significant improvement (*p* = 0.002) in glycated haemoglobin, with a mean difference of −0.18 (95% CI, −0.29, −0.06) in the dietary fibre group compared with placebo, although differences between the two groups were not significant (*p* > 0.05) in relation to fasting blood glucose and homeostatic model assessment of insulin resistance (HOMA-IR) [1]. The two groups were not significantly different (*p* > 0.05) in the subjects who reported adverse events.

The promotion of gut microbes which are SCFA producers in greater diversity and abundance by dietary fibre may be responsible for the improvement in glycated haemoglobin, which could be due in part to increased glucagon-like peptide-1 (GLP-1) production. There is evidence that Bifidobacterium lactis could increase glycogen synthesis, decrease expression of hepatic gluconeogenesis genes, improve translocation of glucose transport-4, and promote glucose uptake [1]. Furthermore, the reduction in body weight of participants in the intervention group compared with control is another area of interest, as this may have contributed to the observed improvement in glycated haemoglobin [1]. Ojo et al. [1] concluded that dietary fibre can significantly improve (*p* < 0.05) the relative abundance of Bifidobacterium, total SCFAs, and glycated haemoglobin. However, the authors observed that dietary fibre did not appear to have significant effect (*p* > 0.05) on fasting blood glucose, HOMA-IR, acetic acid, propionic acid, butyric acid, and adverse events.

In a review by Salaza et al. [2], it was noted that the dysfunction of the gut microbiota is a contributory factor in the pathophysiology of diabetes. It was also reported that the lipid products derived from the gut microbiota may interact with cells from the immune system, and this may have immunomodulatory effect. In particular, microbiota dysbiosis could cause increased production of lipopolysaccharides leading to damage to the intestinal barrier [2]. The prevailing pro-inflammatory environment can lead to a state of insulin resistance and hyperglycaemia [2]. In contrast, the production of SCFAs during eubiosis is useful in maintaining the integrity of the intestinal barrier [2]. The authors suggested that dysbiosis of the gut microbiota can be reversed through the modification of the eating habits of patients or with the administration of prebiotics, probiotics, and symbiotics.

In a related study, Ren et al. [3] conducted a randomised controlled trial to determine the effect of an almond-based low carbohydrate diet on depression and glycometabolism, gut microbiota, and fasting GLP-1 in patients with type 2 diabetes. Forty-five participants with type 2 diabetes who were recruited from the diabetes club and the Endocrine Division of the First and Second Affiliated Hospital of Soochow University were randomly assigned to a low-carbohydrate diet (LCD) or a low-fat diet (LFD) [3]. The dietary intervention was for a period of 3 months, including 22 participants in the LCD group and 23 in the LFD group. The measures of depression and biochemical parameters such as glycated haemoglobin, gut microbiota, and GLP-1 concentration were assessed at baseline and post intervention. The two groups were then compared. Ren et al. [3] found that LCD significantly improved depression and glycated haemoglobin (*p* < 0.01) and increased SCFAs producing bacteria *Roseburia*, *Ruminococcus*, and *Eubacterium*. In addition, the GLP-1 concentration in the LCD group was higher than that in the LFD group (*p* < 0.05). The authors concluded that LCD may provide beneficial effect on depression and glycometabolism in patients with type 2 diabetes. It was further suggested that the role of LCD in improving depression in patients with type 2 diabetes may be related to its role in stimulating the growth of SCFAs-producing bacteria, increasing SCFA production and further maintaining GLP-1 secretion [3].

McKenzie et al. [4] assessed the effect of type 2 diabetes prevention programme aimed at reducing hyperglycaemia and normalising glycaemia in order to delay or prevent the progression to type 2 diabetes. The study involved 96 patients with prediabetes who received intervention involving carbohydrate restricted nutrition therapy for two years that was delivered through remote care team. It was found that the estimated cumulative incidence of normoglycemia and type 2 diabetes at two years were 52.3% and 3%, respectively [4]. There was significant decrease in the prevalence of metabolic syndrome, obesity, and suspected hepatic steatosis at two years [4].

There is evidence that the inflammation due to gingivitis and/or periodontitis tends to worsen during pregnancy due to hormonal changes, and these have implications for maternal health beyond the oral cavity [5]. For example, this inflammation could lead to a low-grade systemic inflammatory and metabolic disturbances which can have an impact on pregnancy outcomes [5]. Therefore, there appears to be a relationship between periodontal inflammation and diabetes, and thus managing inflammatory periodontal diseases may improve glycaemic control and associated diabetic complications. In recognition of this, Rodrigues Amorim Adegboye et al. [5] conducted a randomised controlled trial to explore the effect of a non-pharmacological multi-component intervention on periodontal health and metabolic and inflammatory profiles among pregnant women with periodontitis. The study involved 69 pregnant women receiving prenatal care in a Brazilian public health centre who were randomly allocated to four groups: (1) fortified sachet (vitamin D and calcium) and powdered milk plus periodontal therapy during pregnancy (early PT), (2) placebo sachet and powdered milk plus early PT, (3) fortified sachet and powdered milk plus late PT (after delivery), and (4) placebo sachet and powdered milk plus late PT [5]. The mean bleeding on probing (BOP) was significantly reduced in the early PT groups, while BOP worsened in the late PT groups. No significant effect of fortification on BOP was observed, and there were also no significant differences in the glucose levels and birthweight among the groups [5].

The use of dietary interventions such as dietary fibre and low carbohydrate diet in modulating gut microbiota dysbiosis, promoting SCFAs production and glycaemic control in patients with type 2 diabetes is evident. However, as this is an evolving area of research, more studies are needed to further understand the mechanism of the role of diets on gut microbiota.

## Data Availability

No new data were created or analyzed in this study.

## References

[1] Ojo O., Feng Q.-Q., Ojo O.O., Wang X.-H. (2020). The Role of Dietary Fibre in Modulating Gut Microbiota Dysbiosis in Patients with Type 2 Diabetes: A Systematic Review and Meta-Analysis of Randomised Controlled Trials. Nutrients.

[2] Salazar J., Angarita L., Morillo V., Navarro C., Martínez M.S., Chacín M., Torres W., Rajotia A., Rojas M., Cano C. (2020). Microbiota and Diabetes Mellitus: Role of Lipid Mediators. Nutrients.

[3] Ren M., Zhang H., Qi J., Hu A., Jiang Q., Hou Y., Feng Q., Ojo O., Wang X. (2020). An Almond-Based Low Carbohydrate Diet Improves Depression and Glycometabolism in Patients with Type 2 Diabetes through Modulating Gut Microbiota and GLP-1: A Randomized Controlled Trial. Nutrients.

[4] McKenzie A.L., Athinarayanan S.J., McCue J.J., Adams R.N., Keyes M., McCarter J.P., Volek J.S., Phinney S.D., Hallberg S.J. (2021). Type 2 Diabetes Prevention Focused on Normalization of Glycemia: A Two-Year Pilot Study. Nutrients.

[5] Rodrigues Amorim Adegboye A., Dias Santana D., Teixeira dos Santos P.P., Guedes Cocate P., Benaim C., Trindade de Castro M.B., Maia Schlüssel M., Kac G., Lilienthal Heitmann B. (2021). Exploratory Efficacy of Calcium-Vitamin D Milk Fortification and Periodontal Therapy on Maternal Oral Health and Metabolic and Inflammatory Profile. Nutrients.

